# Clinical Utility of Whole Exome Sequencing and Targeted Panels for the Identification of Inborn Errors of Immunity in a Resource-Constrained Setting

**DOI:** 10.3389/fimmu.2021.665621

**Published:** 2021-05-21

**Authors:** Clair Engelbrecht, Michael Urban, Mardelle Schoeman, Brandon Paarwater, Ansia van Coller, Deepthi Raju Abraham, Helena Cornelissen, Richard Glashoff, Monika Esser, Marlo Möller, Craig Kinnear, Brigitte Glanzmann

**Affiliations:** ^1^ SAMRC Centre for Tuberculosis Research, DSI-NRF Centre of Excellence for Biomedical Tuberculosis Research, Division of Molecular Biology and Human Genetics, Faculty of Medicine and Health Sciences, Stellenbosch University, Cape Town, South Africa; ^2^ Immunology Unit, Division of Medical Microbiology, National Health Laboratory Service and Faculty of Medicine and Health Sciences, Stellenbosch University, Tygerberg Hospital, Cape Town, South Africa; ^3^ Department of Paediatrics and Child Health, Faculty of Medicine and Health Sciences, Stellenbosch University, Tygerberg Hospital, Cape Town, South Africa; ^4^ Division of Haematopathology, National Health Laboratory Service, Faculty of Medicine and Health Sciences, Stellenbosch University, Tygerberg Hospital, Cape Town, South Africa; ^5^ SAMRC Genomics Centre, Cape Town, South Africa

**Keywords:** inborn errors of immunity, South Africa, whole exome sequencing, targeted sequencing, genetic variants

## Abstract

Primary immunodeficiency disorders (PIDs) are inborn errors of immunity (IEI) that cause immune system impairment. To date, more than 400 single-gene IEI have been well defined. The advent of next generation sequencing (NGS) technologies has improved clinical diagnosis and allowed for discovery of novel genes and variants associated with IEI. Molecular diagnosis provides clear clinical benefits for patients by altering management, enabling access to certain treatments and facilitates genetic counselling. Here we report on an 8-year experience using two different NGS technologies, namely research-based WES and targeted gene panels, in patients with suspected IEI in the South African healthcare system. A total of 52 patients’ had WES only, 26 had a targeted gene panel only, and 2 had both panel and WES. Overall, a molecular diagnosis was achieved in 30% (24/80) of patients. Clinical management was significantly altered in 67% of patients following molecular results. All 24 families with a molecular diagnosis received more accurate genetic counselling and family cascade testing. Results highlight the clinical value of expanded genetic testing in IEI and its relevance to understanding the genetic and clinical spectrum of the IEI-related disorders in Africa. Detection rates under 40% illustrate the complexity and heterogeneity of these disorders, especially in an African population, thus highlighting the need for expanded genomic testing and research to further elucidate this.

## Introduction

Primary immunodeficiency disorders (PIDs) are a group of genetically heterogeneous inborn errors of immunity (IEI), which lead to a predisposition to the development of autoimmune and inflammatory diseases, lymphoproliferation and infection (1–5). Individually, IEIs are rare, but collectively represent a significant burden of disease (4, 6). It has recently been estimated that close to one million people in Africa suffer from an IEI, with South Africa contributing approximately 42,000 cases (7). However, human immunodeficiency virus (HIV), tuberculosis (TB) and other infectious epidemics in sub-Saharan Africa, limited clinical and laboratory infrastructure for IEIs, as well as lack of awareness for IEIs in these settings, have been major obstacles to diagnosis and treatment (7–10).

The diagnostic workup for a suspected IEI is shifting from only detailed clinical evaluations of the patient, to include a number of laboratory tests which may include targeted gene screening and immunological assays. Given that most IEIs are monogenic, genetic testing has become an important modality for providing an accurate and definitive diagnosis, altering management and aiding genetic counselling (11–13). This said, the identification of a disease-associated variant in a suspected case of IEI can be challenging with many cases having no single causative variant identified and large genotype-phenotype variation reported (14–16).

The identification of genes and genetic variants associated with IEIs has increased exponentially, with International Union of Immunological Societies (IUIS) currently reporting on 406 distinct disorders, with 430 genetic defects (6). Between 2015 and 2017, a total of 85 new genes were associated with IEIs (17). This increase has largely been driven by the use of next generation sequencing (NGS), more specifically whole genome sequencing (WGS), whole exome sequencing (WES) and the more restricted targeted gene panel sequencing (17). WES involves the investigation of all the protein-coding regions of the human genome (exome) allowing identification of around 85% of all disease-associated mutations (18). NGS gene panels are more targeted and analyse only known disease-associated genes for specific diseases. The advantages of panels over WES or WGS include lower cost, simpler analysis, and optimisation of variant detection in the included genes. Disadvantages include the inability to analyse or re-analyse genes not included on the panel (19).

Currently, NGS gene panels are the mainstay diagnostic tools in clinical settings, whereas WES and WGS may be second-line testing options. In South Africa, WES and WGS largely remain research tools because of their cost and the complexity of data interpretation. Factors that limit their provision in clinical service as diagnostic/clinical tests are that they must have acceptable analytical and clinical validity and clinical utility (20), must meet rigorous quality assurance standards, and must ensure rapid turnaround times in order to achieve ethical test outcomes (20). In a developing country, clinical genomic testing remains a considerable logistical challenge, with low volumes of samples, from patients with rare genetic diseases, leaving them to be considered lower priority.

In the interests of patient care, Tygerberg Hospital has sourced clinical genomic tests from specific international genomic testing providers since 2017, and supplemented this with research-based WES/WGS, at no cost to the patient. To date more than 730 diagnostic panels have been ordered across all clinical areas, of which 28 have been ordered on patients with suspected IEIs. WES has been performed on the DNA of 54 patients, two of whom had prior NGS panel testing.

The benefit of using NGS in patients with suspected IEIs has been shown in numerous international studies (11, 16, 21–23) and is likely to be beneficial in African patients. However it is likely that pathogenic variants, and perhaps even genes, may differ in the African population compared to more intensively studied populations. There was therefore a need to assess the clinical utility of gene panel testing and/or WES in a South African cohort. We report on molecular results, diagnoses and alterations in clinical management in patients with suspected IEIs, tested within the South African state healthcare system.

## Materials and Methods

### Patients

The study includes 80 patients and 107 family members recruited over an 8 year period (2013-2020). Most of the patients have received or are currently receiving their medical care at Tygerberg Hospital, a state academic hospital situated in Cape Town that provides tertiary level services to a population of nearly 3 million people in the Western Cape province of South Africa. The immunology service also receives referrals from further afield in South and southern Africa. Three patients included in this study are receiving medical care in the private healthcare sector and one was within the Gauteng state healthcare system. These patients’ data were included in this study as the families had either sought care from one of the immunology or genetic healthcare specialists working at Tygerberg Hospital or had received genetic testing through this system.

WES was performed in-house on DNA samples from 54 patients. Patients recruited for WES were children between the ages of 0 and 15 years that have a history of severe, unusual, persistent and/or recurrent TB or were suspected to have mendelian susceptibility to mycobacterial disease (MSMD). Depending on the available family members, genomic analysis of a trio (patient and both parents), duo (patient and one parent) or a single individual was performed. Of note is that not all 107 recruited family members were subjected to WES or genomic analysis as a result of either lack of budget or DNA that did not meet quality metrics for sequencing.

NGS diagnostic panel testing became available in 2017 through an external provider, and 28 gene panel tests were performed. Gene panel testing was offered to patients thought to have any one of a broad range of IEIs. This was done following a clinical and immunological assessment by a rheumatologist/clinical immunologist, and assessment of the family history by a genetic counsellor. Tygerberg hospital budget funded panel testing for 22 of the 28 patients, and this was performed through Invitae Corporation (San Francisco, California, United States). Panel selection was based on the clinical phenotype and presentation of the patient, with the number of genes available for inclusion increasing from 207 to 407 genes over time. In January 2019, a number of IEI diagnostic gene panel tests were made available free of charge through a pilot sponsored programme for Jeffrey Modell Centers Network (JMCN) patients, in collaboration with Invitae Laboratories. This allowed access to a 207 gene panel for 6 patients who were not eligible for tests through the hospital and could not themselves afford the cost (2, 4, 17). For patients who received NGS panel tests, family member testing was performed, if required for resolution of variants of uncertain significance (VUS) or for carrier testing of relatives.

NGS gene panels soon became the first-line genomic diagnostic test, with patients that had a negative panel result, or an atypical clinical phenotype, then receiving WES if the research budget allowed. Two patients received both panel testing and subsequent WES testing.

### Panel Testing

Saliva was collected for each of the patients in saliva kits provided by Invitae. Saliva was collected and stored in Oragene™ assisted saliva collection kits (DNA Genotek, Ottawa, Ontario, Canada) (OG575/ODG575), specifically for young children or patients where active saliva donation may be difficult. Two saliva tubes totalling 0.75ml of saliva were collected per kit. Saliva was subsequently stored at room temperature and shipped to Invitae for sequencing and analysis of 207 genes well established to be associated with IEIs. Genomic DNA obtained from the submitted samples was enriched for targeted regions using a hybridization-based protocol, and sequenced using Illumina technology. All targeted regions were sequenced with ≥50x depth or in cases where this falls lower than the accepted range, are supplemented with additional analysis. Reads were aligned to reference sequence hg19 (GRCh37). Promoters, untranslated regions, and other non-coding regions were not interrogated. More information on Invitae’s assays can be found here: (https://www.invitae.com/en/assay/).

### Whole Exome Sequencing

DNA was extracted from whole blood of recruited patients and, wherever possible, their parents and additional family members using standard protocols. A total of 5ml of blood was drawn into BD Vacutainer^→^ EDTA blood collection tubes (Becton, Dickinson and Company, Franklin Lakes, New Jersey, United States). DNA was extracted using the Macherey-Nagel NucleoSpin XL DNA extraction kit (GmbH & Co. KG, Düren, Germany). Library preparation was conducted using the Ion AmpliSeq™ Exome RDY Kit and the Ion Xpress™ Barcode Adaptors 1–16 Kit (Life Technologies, Carlsbad, California, United States). The DNA template was prepared on the Ion Chef system using the Ion PI™ Hi-Q™ Chef Kit and the Ion PI™ Chip Kit v3. Sequencing was performed at the Central Analytical Facility (CAF) at Stellenbosch University, Stellenbosch, South Africa using the Ion Proton™ (Thermo Fisher, Carlsbad, California, United States). The average coverage for WES was 30X across the entire exome.

*Variant calling:* Sequences were aligned to the human reference genome, GRCh37 using TMA (version 5.2.1) in the ion-analysis workflow on the Torrent Suite (version 5.2.1). The Variant Caller (version 5.2.1.1) plugin on the Torrent suite was used for base quality score recalibration, indel realignment and variant calling and variant called format (VCF) files were produced. Variant annotation was done using ANNOVAR (5) and variant prioritization was carried out using a custom-designed, in-house method, TAPER™ (21).

*Sanger Sequencing:* All candidate variants identified through WES were verified using Sanger sequencing. Each amplicon was bi-directionally sequenced using the BigDye^®^ Terminator v3.1 Cycle Sequencing Kit (Perkin-Elmer, Applied Biosystems Inc., Foster City, California, USA.), followed by electrophoresis on an ABI 3130XL Genetic Analyzer (Perkin-Elmer, Applied Biosystems Inc., Foster City, California, USA). All automated DNA sequencing reactions were performed at the Central Analytical Facility (CAF) at Stellenbosch University, Stellenbosch, South Africa.

*Variant interpretation*: Variants detected by NGS testing were all rigorously subjected to the American College of Medical Genetics and Genomics and the Association for Molecular Pathology (ACMG-AMP) variant classification criteria in order to establish pathogenicity (24). Variants that were classified as benign or likely benign were not reported in this study. Variants classified as uncertain, likely pathogenic and pathogenic (LP/P) were included.

*Confirmation of diagnosis and reporting:* A WES-based molecular diagnosis was made in a patient when a LP/P variant was found in a gene associated with the phenotype, consistent with the known patterns of inheritance associated with that gene, and confirmed on Sanger sequencing. In these cases, a report was issued to the referring clinician and explained to the patient and their family members by a genetic counsellor.

### Ethics and Institutional Approvals

NGS gene panels were performed as part of clinical diagnostic investigation of patients suspected to have a broad range of IEIs. Tests were preceded by genetic counselling and subject to informed consent. WES was performed as part of two research studies that aimed at identifying genes and variants associated with a specific sub-group of IEIs known MSMD.

The WES studies were approved by the Health Research Ethics Committee of Stellenbosch University (study numbers: N13/05/075 and N13/05/075(A) and done through the Division of Molecular Biology and Human Genetics, Faculty of Medicine and Health Sciences, Stellenbosch University. Patients included in these studies are children between the ages of 0 and 15 years clinically suspected to have MSMD. Such patients were identified by both the National Health Laboratory Services and Desmond Tutu Centre for Tuberculosis and recruited by a clinical immunologist and a clinical geneticist at Tygerberg Hospital. Written and informed consent was obtained from the parents or legal guardians of eligible patients. Children of age were also asked to assent to the study. The studies adhered to the ethical guidelines as set out in the “Declaration of Helsinki, 2013” (16).

## Results

This study includes a total of 80 patients. Patient demographic data can be found in **Table 1**.

**Table 1:** Patient demographic data.

Total number of patients	80
Males	48
Females	32
Age (Mean)	6 years 7 months

A molecular result that explained all or some of the patient’s phenotype was made in 24/80 (30%) cases, using panel and/or WES. Of the patients who had panel testing only, a diagnosis was made on 12/26 (46%). For patients that had WES only, 12/52 (23%), had a confirmed molecular diagnosis. Of the two patients who had both panel and WES, a molecular diagnosis that confirmed part of the phenotype was made by WES in one. A total of 35/80 (44%) patients have uncertain results and 21/80 (26,2%) patients had no variants of interest identified.

### Genetic Spectrum and Clinical Implications of Molecular Findings

In the 24 probands with a confirmed molecular result, 24 different LP/P variants were identified in 19 genes well known to be associated with IEIs. Clinical and molecular information for each proband can be found in **Table 2**. In 22 probands with an uncertain result, VUSs identified using WES will be subject to further investigation using functional studies, family studies (where possible) and follow-up for re-analysis of variant classification over time. This is because they were identified to potentially be causative of the patients’ phenotype, but currently do not meet ACMG-AMP classification criteria to be classified as likely pathogenic or pathogenic (information in **Supplementary Table 1**) (24).

**Table 2:** Clinical presentation of patients with genes with pathogenic/likely pathogenic variants.

ID	Sex	Age at diagnosis	Relevant family history	Main clinical features	Panel/WES	Implicated gene	Variant classification (zygosity)	Accession numbers for pathogenicity scores	Diagnosis	Status	Medical interventions as indicated and/or substantiated by molecular diagnosis
Patients with likely pathogenic/pathogenic variants in genes previously associated with IEI (IUIS criteria)											
001	F	2 years 5 months	None	Recurrent URTI; lymphadenitis; recurrent abscesses of lymph nodes; soft dysmorphism.	Panel^1^	*PIK3R1* c.1425+1G>A (Splice donor)	Pathogenic (heterozygous)	rs587777709/VCV000372467.8	Autosomal dominant activated PI3K-delta syndrome	Alive	Prophylactic antibiotics.
											Annual surveillance for malignancy (Lymphoma).
											Regular surveillance for TB.
											Monthly Immune replacement therapy for life.
											Annual audiology screening.
002	M	8 months	Unknown	Recurrent and severe URTI requiring ICU admission in 1st year of life.	Panel^1^	*CD40LG* c.346G>T p.Gly116Cys	Likely pathogenic (hemizygous)	Invitae internal calling.	CD40 Ligand deficiency/X-linked Hyper IgM syndrome	Demised after molecular diagnosis	3 weekly immune replacement therapy.
											Regular neutrophil screening for indication of GCSF.
											Cotrimoxazole prophylaxis.
											Would have qualified for HSCT.
003	M	4 months	Unknown	Persistent hypoglycemia; eczema; recurrent fevers and infections with persistent neutropenia. Massive hepatosplenomegaly.	Panel^1^	*SLC37A4* c.59G>A p.Gly20Asp; c.1047_1059delinsGGCTAT p.Phe349Leufs*52	Pathogenic (compound heterozygous)	rs193302881/VCV000551776.1 and Invitae internal calling.	Autosomal recessive glycogen storage disease type Ib	Alive	GCSF administration for neutropenia.
											High calorie diet to prevent hypoglycemic attacks.
											Regular infection screening.
											Endocrine surveillance.
											Organ transplant if needed.
004	M	10 months	Mom is a confirmed carrier	Septicaemia with empyema at 8/12, progressive neurodevelopmental delay and encephalopathy with persistent enteroviral shedding from stool, suspected to be oral Polio vaccine derived strain.	Panel^2,3,4^	*BTK* c.215dupA (p.Asn72Lysfs*13)	Pathogenic (hemizygous)	rs886041148/VCV000279713.4	X-linked Agammaglobulinemia	Demised after molecular diagnosis	Ig replacement therapy.
005	F	8 months	No	Recurrent URTI; flat diffuse warts; raised IgE levels.	Panel^5^	*DOCK8* c.3803del (p.Phe1268Serfs*3)	Pathogenic (homozygous)	Invitae internal calling.	Autosomal recessive hyper-IgE syndrome with combined immunodeficiency	Alive	Prophylactic antibiotics.
											Malignancy surveillance of skin lesions.
											Qualifies for HSCT.
006	M	1 year 1 month	Affected maternal uncles (deceased) Mom is a confirmed carrier	Recurrent sepsis and shock; CMV induced LRTI; candida UTI; chronic gastroenteritis; FTT.	Panel^6^	*CD40LG* Deletion (Exon 3)	Pathogenic (hemizygous)	Invitae internal calling.	CD40 Ligand deficiency/X-linked Hyper IgM syndrome	Demised after molecular diagnosis	May have benefited from HSCT.
											Ig replacement therapy.
007	M	1 year 2 months	None	Progressive paralysis likely due disseminated oral polio vaccine; progressive weakness; nosocomial sepsis; reduced CD4 cells, with normal number of C8, C19 and CD16/CD56 cells.	Panel^6^	*RFX5* c.367_368del (p.Leu124Cysfs*21)	Pathogenic (homozygous)	rs1228361094	Bare Lymphocyte syndrome 2	Demised before molecular diagnosis	May have benefited from HSCT.
008	M	2 months	Yes; brother died at 3 months from severe infection (suspected SCID)	SCID; lung disease; CMV; hepatitis.	Panel^1^	*IL2RG* c.116-1G>A (Splice acceptor)	Pathogenic (hemizygous)	rs104895462/VCV000004696.4	X-linked severe combined immunodeficiency	Demised after diagnosis	Ig replacement therapy.
											Did not meet criteria for HSCT due to disseminated persistent CMV infection).
009	F	1 year 8 months	No	PJP in early infancy, hypogammaglobulinemia with normal CD19, later onset neutropenia.	Panel^1^	*MAP3K14* c.1033G>A p.Val345Met	Likely pathogenic (homozygous)	Invitae internal calling	MSMD	Alive	Ig replacement therapy.
											Prophylactic antibiotics.
											Live BCG vaccines avoided in sib at birth. Sibling vaccinated once results confirmed to be normal.
010	F	3 years 3 months	None	Boggy tenosynovitis of wrists and ankles; uveitis	Single gene screen	*NOD2* c.1000C>T p.Arg334Trp	Pathogenic (heterozygous)	rs104895462/VCV000004696.4	Blau Syndrome	Alive	Methotrexate.
											Regular Follow up for uveitis progression.
011	F	2 years 1 month	None	Recurrent septicaemia, oral ulcers; congenital neutropenia; FTT.	Single gene screen	*ELANE* c.416C>T (p.Pro139Leu)* *	Pathogenic (heterozygous)	rs137854448/VCV000016743.4	Severe Congenital Neutropenia	Unknown Patient lost to follow up.	GCSF subcutaneous injections.
											Monitor for malignancies.
012	M	2 years 2 months	Mom is a confirmed obligate carrier	Agammaglobulinemia; absence of mature B-cells; recurrent pneumonias.	Single gene screen	*BTK* Partial Deletion (Exon 11)	Pathogenic (hemizygous)	Novel; Not reported in population databases; Not reported in literature.	X-linked Agammaglobulinemia	Alive	Ig replacement therapy.
											Eligible for gene therapy.
013	M	3 months	Yes: brother died from the same condition; further history of CID in cousins. Both parents are carriers	Multi-lobular pneumonia; low IgG; dysmorphism; diarrhoea.	WES	*TTC37* c.4507 C>T p.R1503C	Pathogenic (homozygous)	rs200067423/VCV000287653.5	Trichohepatoenteric syndrome	Demised before molecular diagnosis	Parenteral nutrition.
											Ig replacement therapy.
											Surveillance for liver dysfunction.
014	F	9 years and 6 months	Yes, affected sibling	Central nervous system and skin; vasculitis, stroke with hemiparesis, seizures, progressive contractures of interphalangeal joints without bone resorption.	WES	*SAMHD1* c.1681_1682del p.Ser561Phe fs*61)	Pathogenic (homozygous)	Novel; Not reported in population databases; Not reported in literature.	Aicardi-Goutières syndrome-5	Alive	Anticoagulant therapy.
											Immunomodulation therapy.
											Surveillance for unusual infections including TB.
											Surveillance for glaucoma.
015	F	14 years	Yes, affected sibling	Contractures of interphalangeal joints without bone resorption, severe glaucoma.	WES	*SAMHD1* c.1681_1682del p.Ser561Phe fs*61	Pathogenic (homozygous)	Novel; Not reported in population databases; Not reported in literature.	Aicardi-Goutieres syndrome-5	Alive Follow up defaulter	Management of glaucoma.
											Surveillance for unusual infections including TB.
016	M	2 years 1 month	Unknown	Recurrent pneumonia and recurrent gastroenteritis. Chronic oral herpes lesions.	WES	*TNFRSF13B* c.236 A>G p.Tyr79Cys	Pathogenic (heterozygous)	rs72553876/VCV000281110.4	Common variable immunodeficiency-2 Normal B and T cells with Low IgG.	Alive	Ig replacement therapy for life.
017	F	9 years 5 months	No	Recurrent bacterial septicemias; pneumonias and herpes *Labialis Molluscum*; encephalitis; intellectual disability.	WES	*LRBA* c.3407 C>T p.Pro1136Leu	Pathogenic (homozygous)	rs113022115/VCV000287734.2	Common variable immunodeficiency-8 with autoimmunity	Demised before molecular dx. Molecular diagnosis made post mortem	Ig replacement therapy and immune modulation eg. CTLA-4 (Orencia) HSCT would have been indicated.
018	F	3 years 7 months	None	Initial cutaneous BCG abscess and later chronic CNS BCG dissemination, severe hypogammaglobulinemia.	WES	*MAP3K14*	Likely pathogenic (homozygous)	VCV000843423.2	CVID and Mendelian susceptibility to mycobacterial disease	Alive	TB surveillance.
						c.1033 G>A					Antibiotic prophylaxis.
						p.Val345Met					Ig replacement therapy.
						Avoid live vaccines.				
019	M	Birth	Yes; male sibling with SCID died in early infancy prior to HSCT	Asymptomatic severe combined immunodeficiency T-B+NK-, identified on basis of positive family history.	WES	*IL2RG* c.443 T>G p.Leu148Arg	Pathogenic (hemizygous)	Novel; Not reported in population databases; Not reported in literature.	X-linked severe combined immunodeficiency	Alive	HSCT successful.
											Thriving requiring no further intervention.
020	M	4 months	Yes, brother died in early infancy from persistent diarrhea, thrombocytopenia (probable WAS)	Chronic diarrhoea, eczematous skin rashes, CMV pneumonitis, septic arthritis, Group B Streptococcal Septicaemia. Delayed onset thrombocytopaenia.	WES	*WAS* c.397 G>A p.Glu133Lys	Pathogenic (hemizygous)	VCV000870492.1	Wiskott–Aldrich syndrome	Alive	Immune replacement therapy.
											Prophylactic antibiotics
											Surveillance of autoimmunity and thrombocytopenia.
											Qualifies for HSCT but no consent.
021	F	6 years 4 months	None	Disseminated verrucae, chronic otitis media, Herpes Keratitis, bacterial pneumonias and suspected pulmonary tuberculosis.	WES	*DOCK8* c.3912del p.N1267fs	Pathogenic (homozygous)	Novel; Not reported in population databases; Not reported in literature.	Autosomal recessive hyper-IgE syndrome with combined immunodeficiency	Alive	Screening for TB, malignancy (cervical and skin) & hepatic disorders.
											Ig replacement therapy.
											Qualifies for HSCT.
022	M	2 years 4 months	Yes; affected maternal uncle	Stable neutropenia, lymphopenia, hypogammaglobulinemia, pneumonias & upper respiratory infections in infancy and early childhood with spontaneous gradual improvement.	WES	*MSN* c.511C>T p.Arg171Trp	Pathogenic (hemizygous)	rs1057519074/VCV000372154.6	Immunodeficiency-50 (Mild phenotype)	Alive	Immune replacement therapy.
											Prophylactic antibiotics.
											HSCT not indicated because of mild phenotype.
023	F	5 years 4 months	None	Hypogammaglobulinemia, isolated ACTH deficiency, asymptomatic pulmonary infiltrates and canalicular liver function abnormalities.	WES	*NFKB2* c.1288C>T p.Pro430Ser	Likely pathogenic (heterozygous)	rs202001697/VCV000474775.4	DAVID syndrome	Alive	Hormone replacement.
											Ig replacement therapy.
											Surveillance for Liver disease and other endocrine deficiencies.
024	F	7 years 6 months	None	Recurrent pneumonias with bronchiectasis, skin abscesses, scoliosis	WES	*STAT1* c.802 G>T p.Glu268Ter	Likely pathogenic (heterozygous) VUS (heterozygous)	Novel; Not reported in population databases; Not reported in literature.	Hyper IgE syndrome	Alive	Immune replacement therapy.
											Prophylactic antibiotics.
											*ZNF341* c.2167 A>C p.Thr723Pro

Panel^1^, PR08100.02: Invitae Primary Immunodeficiency Panel; Panel^2^, PR08111.02.1: Add-on Hypogammaglobulinemia Genes; Panel^3^, PR08111.02.2: Add-on Common Variable Immunodeficiency Genes; Panel^4^, PR08111.02:Invitae Agammaglobulinemia Panel; Panel^5^, PR08113.01:Invitae Hyper IgE Syndrome Panel; Panel^6^, PR08137.02: Invitae Combined Immunodeficiency (CID) Panel; Panel^7^, PR08143.02:Invitae Mendelian Susceptibility to Mycobacterial Disease Panel; Panel^8^, PR08112.01: Invitae Common Variable Immunodeficiency Panel; Panel^9^, PR08112.01.1: Add-on Genes for Primary Immunodeficiencies That Can Mimic Common Variable Immunodeficiency; Panel^10^, PR08114.01: Invitae Hyper IgM Syndrome Panel; Panel^11^, PR08114.01.1: Add-on Clinically-overlapping Genes; Panel^12^, PR08120.02.01: Add-on Autoimmunity Genes; Panel^13^, PR08120.02: Invitae Autoinflammatory Syndromes Panel; Panel^14^, PR08113.04:Invitae Hyper IgE Syndrome Panel; M, male; F, female; WES, whole exome sequencing; URTI, upper respiratory tract infections; FTT, failure to thrive; UTI, urinary tract infection; TBM, tuberculosis meningitis; ACTH, adrenocorticotropic hormone; LRTI, lower respiratory tract infections; ACTH, adrenocorticotropic hormone; LRTI, lower respiratory tract infections; CID, Combined Immunodeficiency; GIT, gastrointestinal tract; HSCT, haematopoietic stem cell transplant; GCSF, Granulocyte colony-stimulating factor; CMV, Cytomegalovirus; PJP, Pneumocystis jirovecii pneumonia; DAVID, Deficient anterior pituitary with variable immune deficiency; BCG, Bacillus Calmette Guérin; CNS, central nervous system; VUS, variant of unknown significance; ICU, intensive care unit.

For 24 probands with a confirmed molecular diagnosis, the mode of inheritance was found to be autosomal recessive in 10/24 (41,2%) cases; X-linked recessive in 8/24 (33,3%) cases and autosomal dominant (*de novo* or due to germline mosaicism) in 6/24 (25%) cases. Results are summarised in **Table 3**.

**Table 3:** Summary of results from probands and family member testing.

PROBANDS	AR IEI	XLR IEI	AD IEI	TOTAL
Probands with LP/P variant on panel	4 (3 homozygous; 1 compound heterozygous)	5 (hemizygous males)	3 (heterozygous)	12
Probands with LP/P variant on WES	6 (homozygous or compound heterozygous)	3 (hemizygous males)	3 (heterozygous)	12
Probands total	10 (homozygous or compound heterozygous)	8 (hemizygous males)	6 (heterozygous)	24
**FAMILY MEMBERS**	**AR IEI**	**XLR IEI**	**AD IEI**	**TOTAL**
Family members identified as carriers	20 (either through testing or known obligate carrier)	8* (heterozygous females)	0	28
Family member excluded as carriers/affected	n/a	1 (maternal aunt of proband)	6*	7

*All were parents of probands.

VUS, variant of unknown significance; WES, whole exome sequencing; AR, autosomal recessive; XLR, X-linked recessive; AD, autosomal dominant; LP/P, likely pathogenic/pathogenic; IEI, inborn error of immunity.

Molecular diagnosis altered management in 67% (12/18) of living patients. Management could have been altered in 29,1% (7/24) of cases with a molecular diagnosis if referral and diagnosis was made sooner and patients had not died. Having a molecular diagnosis also allowed for family cascade testing, identifying 19 families at risk of having another child affected with an IEI.

## Discussion

NGS is becoming the gold standard for confirming diagnoses in patients with suspected IEIs (12, 15) with our detection rates being comparable to other published studies (14, 16). This study highlights several important aspects of the clinical utility of NGS testing in patients with IEIs, even in setting where the healthcare services are significantly resource-constrained, and the majority of patients belong to indigenous African populations (populations considered understudied in genetic research) (25).

Our results show that NGS testing yielded a definitive diagnosis in 30% (24/80) of patients tested. Three of these diagnoses were made after the patient died and four died shortly after diagnosis was made. In 67% of the 18 living patients, a molecular diagnosis allowed significant alteration to management that in the long term may reduce morbidity, mortality and costs of care. Significant changes to health management included two patients successfully receiving HSCT and three more being eligible for HSCT as a curative option. It is now known that some patients have variant associated malignancy risks and screening for this has been put into place. Definitive diagnosis has also allowed access to various other clinical screenings for some such as audiological, endocrinological and haematological screening, as well as dedicated ophthalmology screening (e.g. for glaucoma and uveitis). Various diagnosed PIDDs are known to benefit from antibiotic prophylaxis based on risks for bacterial infection, and relevant patients will now appropriately receive this either chronically or intermittently. Diagnosis has also assisted with identifying patients that are required to avoid live vaccines because of known risks for dissemination. One patient is now under observation as a candidate for organ transplant (liver) in adulthood. The remainder of live patients with a confirmed genetic diagnosis largely continued on treatment prescribed pre molecular diagnosis. However, having a confirmed genetic result allows them to potentially be eligible for curative treatments such as gene therapy in the future.

A total of 5 patients with life-threatening IEIs in this cohort died before receiving HSCT as a curative therapy, highlighting the need for rapid diagnosis in IEIs. This issue urgently requires increased awareness of PIDs among primary practitioners and subsequent referral to clinical immunology and genetic services without delay. It also requires early use of NGS testing as part of patient care, where possible in the form of clinical/diagnostic testing (as opposed to research recruitment). In our services, these difficulties are improving over time as awareness increases of the availability of suitable NGS testing, and it becomes more widely understood. These improvements have resulted in part from an active molecular genetic research program in the field of IEIs. In addition, there is hospital buy-in from Tygerberg Hospital to the clinically appropriate use of NGS clinical/diagnostic testing for rare disease diagnosis, but this is not currently the case at national level, and there remains an urgent need for national policy on NGS diagnostic testing for rare diseases.

A positive outcome for all families whether with deceased or living probands is that molecular results allowed for accurate genetic counselling around diagnosis and prognosis and allowed for the provision of psychosocial support. The confirmed results allowed parents and interested extended family members to have carrier testing to better understand recurrence risks. Of relevance to testing in resource constrained environments is that familial cascade testing, for probands who had a positive molecular result on an Invitae Panel, is free of charge.

In this cohort, familial testing allowed for 19 sets of parents to know their carrier status and therefore potential reproductive risks. For the 11 sets of parents that are proven or assumed obligate carriers of an autosomal recessive condition, the risk to have another affected child is 25% for each pregnancy. The 8 mothers that were confirmed to be carriers of an X-linked IEI could be informed of the 50% chance of sons being affected and daughters being carriers. One maternal aunt of a proband with an X-linked IEI was excluded as a carrier. The autosomal dominant conditions in this cohort were all seen to be *de novo* with low recurrence risk for future pregnancies (as germline mosaicism cannot be excluded) and no risk to extended family members. Although not relevant to this cohort, genomic testing to identify asymptomatic affected individuals has an important clinical role in allowing early diagnosis to allow for screening and preventative measures to improve health outcomes (13, 22, 26, 27).

Knowing carrier status provides access prenatal diagnosis (PND), with the option of termination of an affected pregnancy, or to prepare for the birth of an affected child, in which case it facilitates optimal neonatal management (e.g. avoid live vaccinations; haplotyping for a HSCT donor) (28, 29). Although the data are not presented, we are aware that several family members have received PND in subsequent pregnancies. The option of preimplantation genetic diagnosis (PGD) to prevent an affected pregnancy is another consideration, but is unfortunately not available within the South African state health sector. PGD must be accessed privately and requires considerable financial means.

Even with NGS-based approaches dramatically improving IEI diagnoses, detection rates remain below 50% (14–16). Proposed reasons include IEIs with a complex mode of inheritance, limitations of certain genomic technologies and cases caused by genes and variants not yet discovered or understood (5, 14–16, 19). Our results have shown the same with a detection rate of 30%. In consanguineous families, this detection rate may be as high as 70%, indicating the clinical utility of these NGS platforms (30). Lower detection rates in a South African cohort may also be due to limitations of testing methods used. TGPs look only at a specific subset of genes which have been clinically associated with disease, while WES looks at 1,2% of the human genome, specifically that which, when translated and transcribed, will be expressed as proteins. TGP and WES share a major limitation in that both methods rely on an exon-capture step upstream of the sequencing. Various approaches to exon capture have been developed and modified, but no single method is able to ensure perfect uniformity of target coverage – put plainly, some regions may sequence better and at a higher vertical coverage than others (26, 31, 32). Coupled to this, WES does not consider the fact that non-coding regions of the genome harbour an estimated 15% of variants which have large effect sizes on disease traits and for this reason, any pathogenic variant which may be in these regions, would not be identified (26).

Given the restrictiveness of both TGP and WES, numerous variants and/or novel genes may have been missed as a possible cause for IEI in the remaining 70% of the patients. Multiple studies have shown that WGS is more powerful than TGP and WES as this technique expands beyond targeted regions, and is not restricted by the capture methods which is a significant limitation in both TGP and WES (26, 33–35). WGS was not used on patients in this study even though it is the better genomic testing option in rare diseases. Going forward, a number of patients in this study with no definitive diagnosis or candidate genetic variants identified, will be subjected to WGS as a continuation of the research projects earlier described. Patients with IEIs not previously recruited will also be considered for WGS, with the hopes of improving understanding of genetic contribution to IEIs in African patients and in increasing diagnosis.

Lower detection rates in this cohort could also be attributed to genomic testing in a population in which the genome is highly diverse, understudied and poorly understood. In African patients, undiscovered variants and their corresponding immune pathways may account for differences in susceptibility and consequent clinical manifestations of disease, making variant calling and interpretation following NGS testing a huge challenge (25, 36). To overcome such limitations, there is an urgent need for more research focusing on African genetic diversity in both health and disease, and how this determines the predominant host specific immune responses (25).

An interesting finding from this study was that detection rates using targeted NGS gene panels were higher than detection rates using broader based WES (46% vs 25%), despite panel testing being more restrictive than WES. This is possibly due to more focused phenotyping of patients selected for panels versus those selected for research-based WES. Patients selected for WES were done so based on history of severe, unusual, recurrent or persistent TB and therefore thought to have MSMD. Using such inclusion criteria in a country where the rates of TB are exceptionally high, may have resulted in including patients with a clinical picture that is attributed to environmental exposures and social circumstances, rather than due to an underlying genetic defect. There also remains the possibility that the underlying genetic defects for susceptibility to mycobacterial disease, such as TB, is different in the African population, making gene and variant prioritisation through current bioinformatic pipelines difficult. Selecting for patients with suspected MSMD remains a challenge in countries, such as South Africa, where TB remains a pandemic and very careful evaluation by immunology and genetics experts is needed to ensure resources are not unnecessarily spent.

This study has highlighted how genomic testing has positively altered clinical and family management in a significant number of patients. Motivation for the government to allocate funds for genomic services and testing going forward requires evidence of clinical and public health utility and this study serves as the first of its kind to show this in South Africa. The challenge remains getting government funding for incorporation of such testing into routine clinical care for patients with suspected IEIs. The rapid decline in NGS sequencing cost and the per-sample cost of a singleton WES (i.e. on the proband only) being comparable to that of targeted panels should convince public and private health funders to consider implementing such testing in funded as well as resource constrained environments. Our findings allow the start of the conversation to mainstream NGS testing for IEIs, with the goal being improved overall patient and family care.

## Data Availability Statement

The datasets presented in this study can be found in online repositories. The names of the repository/repositories and accession number(s) can be found in the article/**Supplementary Material**.

## Ethics Statement

The studies involving human participants were reviewed and approved by Health Research Ethics Committee of Stellenbosch University [N13/05/075 and N13/05/075(A)]. Written informed consent to participate in this study was provided by the participants’ legal guardian/next of kin. Written informed consent was obtained from the minor(s)’ legal guardian/next of kin for the publication of any potentially identifiable images or data included in this article.

## Author Contributions

CE, MU, ME, MM, CK, and BG conceived the project. BP and AC conducted all laboratory experiments. BG conducted all bioinformatics analysis of WES data. MS, CE, and MU assisted with the genetic counselling. ME, DA, and HC were involved with patient recruitment. All authors contributed to the article and approved the submitted version.

## Funding

Research reported in this publication was partially funded by the National Health Laboratory Service Research Trust (GRANT004 94525) and the South African Medical Research Council. The content is the sole responsibility of the authors and does not necessarily represent the official views of the South African Medical Research Council. This work was also supported by the National Research Foundation of South Africa unique grant number 93360 titled “Identifying novel TB susceptibility candidate genes using NGS data from individuals with PIDs”. The funders had no role in study design, data collection and analysis, decision to publish, or preparation of the manuscript.

## Conflict of Interest

The authors declare that the research was conducted in the absence of any commercial or financial relationships that could be construed as a potential conflict of interest.
