# Supplementary material for: Clinical Utility of Whole Exome Sequencing and Targeted Panels for the Identification of Inborn Errors of Immunity in a Resource-Constrained Setting
Source: Front Immunol. 2021 May 21;12:665621. doi: 10.3389/fimmu.2021.665621 (PMC8176954; doi:10.3389/fimmu.2021.665621)
Supplement: Supplementary file 1 [file Table_1.docx]

Supplementary Material

# Supplementary Table

Supplementary Table 1: Clinical presentation of patients with uncertain results.

| **Patient ID** | **Sex** | **Age at diagnosis** | **Relevant family history** | **Main clinical features** | **Panel/ WES** | **Current treatment** | **Candidate gene/variant identified? (Y/N)** | **Status** |
| --- | --- | --- | --- | --- | --- | --- | --- | --- |
| 025 | M | 3 years 2 months | Yes; affected brother | Recurrent URTI | Panel^1^ | Ig replacement therapy | N  (candidate for WGS) | Alive |
| 026 | F | 2 years 3 months | Unknown | Extensive skin rash, HIV infected, clubbed and TB | Panel^1^ | Antiretroviral therapy | N  (candidate for WGS) | Unknown (lost to follow up) |
| 027 | F | 2 years 8 months | None | Primary lymphoedema, secondary T cell lymphopenia & non-invasive skin infection | Panel^1^ | Symptomatic therapy  with compression stockings, meticulous skin care and  and antibiotics prn | N  (candidate for WGS) | Alive |
| 028 | M | 1 year 1 month | None | Staphylococcal pneumonia and septic arthritis, hypogammaglobulinemia with normal CD19 | Panel^1^ | Ig replacement therapy | N  (candidate for WGS) | Alive |
| 029 | M | 2 years 2 months | Dad previously had TB and MDR TB | Recurrent TBM; neurodevelopmental delay | Panel^1,7^ | Suspected MSMD for surveillance of future TB episodes. | N  (candidate for WGS) | Unknown (lost to follow up) |
| 030 | M | 5 months | No | FTT; recurrent, persistent pneumonia,  *Mycobacterium abscessus* infection | Panel^1,7^; WES | Suspected MSMD for surveillance of future TB episodes.  Monitoring growth and development | Y | Alive |
| 031 | F | 49 years 9 months | Unknown | In childhood: Bacterial meningitis, recurrent tonsillitis, measles, mumps, rubella, varicella zoster, annual recurrent respiratory tract infections (bronchitis and pneumonia) with multiple hospital admissions  Adulthood: recurrent pneumonias; agammaglobulinemia and absent vaccine responses but with preserved B cells. | Panel^1,4,8,9^ | Ig Replacement therapy | N | Unknown |
| 032 | M | 9 months | Possible affected father - Hydroadenitis Suppurativa | hypogammaglobulinemia; ectodermal features; low grade IBS; Recurrent URTI. | Panel^1,4,8,9^ | Ig replacement therapy  Prophylactic Antibiotics | Y | Alive |
| 033 | F | 4 years 9 months | No | Orbital Lymphomatoid Granulomatosis (variant of diffuse Large B cell lymphoma Grade III); Post Chemo : Secondary Hypogammaglobulinemia (Elevated IgM; low IgA/IgE, Absent IgG, normal CD19 | Panel^1,10,11^ | Ig Replacement therapy  Prophylactic antibiotics  Surveillance for recurrence of Malignancy | N  (candidate for WGS) | Alive |
| 034 | M | 2 years 6 months | No | MDR TBM, polyarthritis, uveitis with  blindness, aortic aneurysms due to vasculitis | Panel^1,12,13^ | Suspected MSMD, for surveillance of future TB    Immunosuppression (Methotrexate and Humira) | N  (candidate for WGS) | Alive |
| 035 | F | 10 years 8 months | No | Persistent pulmonary TB unresponsive to 6 months of standard therapy. | Panel^1,7^ | Extended inpatient TB treatment  Suspected MSMD for surveillance of future TB | N  (candidate for WGS) | Alive |
| 036 | F | 3 years 4 months | Unknown | Severe persistent disseminated tuberculosis with hepatomegaly and cervical adenopathy | Panel^1,7^ | Suspected MSMD for surveillance of future TB | N  (candidate for WGS) | Alive |
| 037 | F | 4 years 3 months | No | Suspected Autoinflammatory disorder with recurrent infections;  hypertriglyceridemia and hypercholesterolemia, mild lipodystrophy. | Panel^1^ | Immunosuppression with steroids, Methotrexate and Azathioprine.  Fat free diet.  Regular screening for infection, liver derangements and lipogram studies. | N  (candidate for WGS) | Alive |
| 038 | M | 1 year 1 month | No | Recurrent URTI; Eczema, Elevated IgE and eosinophils, HIES score >40 | Panel^14^ | Prophylactic antibiotics  Bronchodilators  HPV vaccine  Regular follow up | N  (candidate for WGS) | Alive |
| 039 | M | 8 years | No | Autoinflammatory syndrome with Periodic fever pattern without infection; abdominal pain attacks; responsive to colchicine | Panel^12,13^ | Colchicine | N | Alive |
| 040 | F | 9 years 1 month | No | Recurrent and unusual TB, including MDR TB | WES | Suspected MSMD for surveillance of future TB episodes  Prophylactic INH | Y | Alive |
| 041 | M | 10 years |  | Recurrent cold abscesses; recurrent and unusual pulmonary TB | WES | Suspected MSMD for surveillance of future TB episodes | Y | Alive |
| 042 | M | 4 years and 1 month | No | Recurrent lymphadenitis and pulmonary TB | WES | Suspected MSMD for surveillance of future TB episodes | Y | Alive |
| 043 | F | 2 years and 2 months | No | Disseminated TB (pulmonary and abdominal TB) | WES | Suspected MSMD for surveillance of future TB episodes | Y | Unknown |
| 044 | F | 4 years | No | Features of Hyper IgE related syndrome with normal IgE levels. | WES | Surveillance for   malignancies and infections including TB  HPV vaccine | Y | Alive |
| 045 | F | 10 years and 6 months | No | Recurrent pulmonary TB and lymphadenitis. One episode of MDR TB | WES | Suspected MSMD for surveillance of future TB episodes | Y | Alive |
| 046 | F | 33 years | No | Unusual and recurrent rib and spinal TB | WES | Suspected MSMD for surveillance of future TB episodes | Y | Unknown |
| 047 | F | 10 years and 4 months | No | Severe TBM and pulmonary TB. | WES | Suspected MSMD for surveillance of future TB episodes | Y | Alive |
| 048 | M | 3 months | No | Empyema, *Mycobacterium abscessus* cultured on gastric washes | WES | Suspected MSMD for surveillance of future TB episodes | Y | Unknown |
| 059 | M | 5 years and 4 months | No | Recurrent pulmonary TB | WES | Suspected MSMD for surveillance of future TB episodes | Y | Alive |
| 050 | M | 3 years | No | Recurrent and severe TB | WES | Suspected MSMD for surveillance of future TB episodes | Y | Alive |
| 051 | M | 13 years | No | Unusual Extrapulmonary TB : Spine, Hepar, para-aortic granulomas | WES | Suspected MSMD for surveillance of future TB episodes | Y | Unknown |
| 052 | M | 7 months | Yes; 4th deceased infant (Brain atrophy) | Recurrent URTI, gastroesophageal reflux disease | WES | Suspected MSMD for surveillance of future TB episodes | Y | Demised |
| 053 | F | 1 year 3 months | No | Granulomatous disease with skin rash, erythema nodosum, generalized lymphadenopathy & hepatosplenomegaly , severe uveitis with bilateral blindness | WES | Immunomodulatory and Immunosuppressive therapy (Infliximab, methotrexate, steroids) | Y | Alive |
| 054 | M | 6 years | Yes, uncle and dad with TB | Recurrent and unusual TB - TB of the spine and TB lymphadenitis | WES | Suspected MSMD for surveillance of future TB episodes | Y | Unknown |
| 055 | M | 14 years 5 months | None | Recurrent TB, and clinical Shprintzen-Goldberg syndrome (marfanoid habitus, craniosynostosis, learning disability). | WES | Suspected MSMD for surveillance of future TB episodes | Y | Unknown |
| 056 | F | 11 years | Unknown | Recurrent, unusual TB; multiple cold abscesses and TB lymphadenitis - inguinal lymph nodes | WES | Suspected MSMD for surveillance of future TB episodes | Y | Alive |
| 057 | F | 10 years 6 months | No | Recurrent/persistent TB, unusual TB (liver); intermittent fever, vomiting and a distended abdomen (hepatomegaly) | WES | Suspected MSMD for surveillance of future TB episodes | Y | Unknown (lost to follow up) |
| 058 | F | 5 years 3 months | None | Severe (disseminated) and recurrent TB; unusual TB (TB osteitis) | WES | Suspected MSMD for surveillance of future TB episodes | Y | Unknown |
| 059 | M | 10 months | Brother with similar symptoms/features | Recurrent viral infections including CMV, HSV, dysmorphism; eczema; FTT; neurodevelopmental delay; hepatosplenomegaly | WES | Ig replacement therapy | Y | Unknown (lost to follow up) |

Panel^1^: PR08100.02: Invitae Primary Immunodeficiency Panel; Panel^2^: PR08111.02.1: Add-on Hypogammaglobulinemia Genes; Panel^3^: PR08111.02.2: Add-on Common Variable Immunodeficiency Genes; Panel^4^: PR08111.02:Invitae Agammaglobulinemia Panel; Panel^5^:  PR08113.01:Invitae Hyper IgE Syndrome Panel;  Panel^6^: PR08137.02: Invitae Combined Immunodeficiency (CID) Panel; Panel^7^: PR08143.02:Invitae Mendelian Susceptibility to Mycobacterial Disease Panel; Panel^8^: PR08112.01: Invitae Common Variable Immunodeficiency Panel; Panel^9^: PR08112.01.1: Add-on Genes for Primary Immunodeficiencies That Can Mimic Common Variable Immunodeficiency; Panel^10^: PR08114.01: Invitae Hyper IgM Syndrome Panel; Panel^11^: PR08114.01.1: Add-on Clinically-overlapping Genes; Panel^12^: PR08120.02.01: Add-on Autoimmunity Genes; Panel^13^: PR08120.02: Invitae Autoinflammatory Syndromes Panel; Panel^14^: PR08113.04:Invitae Hyper IgE Syndrome Panel; M: male; F: female; URTI: upper respiratory tract infections; FTT: failure to thrive; UTI: urinary tract infection; TBM: tuberculosis meningitis; ACTH: adrenocorticotropic hormone; LRTI: lower respiratory tract infections; HIES: Hyperimmunoglobulin E syndrome; MSMD: Mendelian susceptibility to mycobacterial disease; CMV: Cytomegalovirus; HSV: herpes simplex virus.
